# Integrated machine learning–based model and WQI for groundwater quality assessment: ML, geospatial, and hydro-index approaches

**DOI:** 10.1007/s11356-023-25938-1

**Published:** 2023-03-03

**Authors:** Sherif Ahmed Abu El-Magd, Ismael S. Ismael, Mohamed A. Sh. El-Sabri, Mohamed Sayed Abdo, Hassan I. Farhat

**Affiliations:** 1 Geology Department, Faculty of Science, Suez University, Suez, 43518 Egypt; 2 Desert Research Center, Mattaria, Cairo, Egypt

**Keywords:** Machine learning model (ML), SVM, WQI, SVM-WQI, Egypt

## Abstract

The demands upon the arid area for water supply pose threats to both the quantity and quality of social and economic activities. Thus, a widely used machine learning model, namely the support vector machines (SVM) integrated with water quality indices (WQI), was used to assess the groundwater quality. The predictive ability of the SVM model was assessed using a field dataset for groundwater from Abu-Sweir and Abu-Hammad, Ismalia, Egypt. Multiple water quality parameters were chosen as independent variables to build the model. The results revealed that the permissible and unsuitable class values range from 36 to 27%, 45 to 36%, and 68 to 15% for the WQI approach, SVM method and SVM-WQI model respectively. Besides, the SVM-WQI model shows a low percentage of the area for excellent class compared to the SVM model and WQI. The SVM model trained with all predictors with a mean square error (MSE) of 0.002 and 0.41; the models that had higher accuracy reached 0.88. Moreover, the study highlighted that SVM-WQI can be successfully implemented for the assessment of groundwater quality (0.90 accuracy). The resulting groundwater model in the study sites indicates that the groundwater is influenced by rock-water interaction and the effect of leaching and dissolution. Overall, the integrated ML model and WQI give an understanding of water quality assessment, which may be helpful in the future development of such areas.

## Introduction

Nowadays, the management of water resources has become of global concern. Water resources management undergoing for major paradigm shift currently, to ensure these natural resources can be sustained for future development. Over the few decades, the study site of El Salhiya east Delta has faced dramatic growth in reclamation projects. El Salhiya area is one of the most important land reclamation projects and industrial zones in the east Delta of Egypt. At present, the study site has major attention for future development that in turn leads to the occurrence of freshwater. Consequently, these new projects and developments will increase the need for continuous water supply for sustainable development. However, agriculture in the study site is largely the dominant sector for the area and Canal cities. Groundwater in the area is considered one of the most important, freshwater resources. Eissa et al. (2016) concluded that freshwater deterioration in arid areas threatens the water supply sustainability. The deltaic deposits (Pleistocene sediments) represent the main water aquifer (Shata and El Fayoumy 1970). Subsequently, understanding the physio-chemical characteristics of the groundwater is critical to appropriately evaluate the quality of groundwater for irrigation. Therefore, to elaborate on the water suitability for irrigation uses, WQI, SVM, and SVM-WQI approaches were applied to sixty-seven groundwater samples. There are various distinctive approaches were applied to calculate and obtained the WQI, based on the aiming utilize (Sener et al. 2017; Hamlat and Guidoum 2018; Suvarna et al. 2020). Among the indices that contributed to groundwater quality evaluation were TDS, EC, magnesium hazard, sodium percentage, sodium residual carbonate, the ratio of sodium absorption, Kelly index, and index of permeability. Recently, many scientific studies were applied the WQI for the evaluation of water quality (Ramesh and Elango 2011; Sarath et al. 2012; Kumar and James 2013; Vetrimurugan et al. 2013; Shehata and El-Sabrouty 2014; Mabrouk et al. 2016; Ahmed et al. 2020; Elsayed et al. 2020; Abu El-Magd et al. 2021; Masoud and Abu El-Magd 2022; Abu El-Magd et al. 2022a, b). However, the implementation of the WQI method in the present work involved calculating the hazard indices. The application of the WQI using water hazard indices with efficiency values and weights for groundwater assessment in such areas has recently been applied.

Consequently, the popular and faster prediction models and techniques are suggested to state in real-time for groundwater quality evaluation under different stresses. Thus, the data-based methods (MLs) may be valuable approaches, as they have been applied to the assessment and prediction of groundwater quality and are relevant models in terms of accuracy. Nevertheless, the assessment and managing groundwater quality prediction is the most outstanding challenge, which needs suggested new approaches to mitigate water deterioration.

Several linear and nonlinear (support vector machines, artificial neural networks, kernel partial least squares) modeling approaches are available for the regression and classification of complex problems (Singh et al. 2004, 2009; Li et al. 2011; Abu El-Magd et al. 2020; Abu El-Magd 2022). Thus, SVMs have successfully been implemented for both classification and regression in various studies (Mucherino et al. 2009; Kavaklioglu 2011; Kovacevic et al. 2010; Yahya et al. 2019). The dataset and independent variables of the model include records that have been gathered over the present work. Support vector machines (SVMs) are a relatively new machine learning approach and essentially a kernel-based procedure that is based on Vapnik–Chervonenkis theory (Vapnik 1998). Recently, SVMs emerged as one of the leading techniques in pattern classification (Pan et al. 2008). As a key advantage of the SVM, model dimensions and estimation errors can simultaneously minimize. Furthermore, SVM has a little prone to over-fitting and has quite a good generalization ability. However, this context demonstrates the use of the integration of the SVM model in forecasting groundwater quality based on seven WQI parameters.

In this work, the SVM and SVM-WQI models were used to examine their efficiency in the assessment of groundwater quality for irrigation. The novelty of the present work is to increase the credibility of the integration between the SVM as a machine learning approach and the statistical methodology (SVM-WQI) in groundwater modeling. However, the hydro-chemical characteristics, as well as groundwater geochemical evolution were identified in this work. The integration of ML technique, hydrochemistry, and hazard indices evaluation is an important context for identifying groundwater sources and geochemical processes controlling water quality. Therefore, the implemented approach model structure is almost stable; thus, the accuracy and efficiency of calculation were high, which makes the applied approach worthy of promotion and application for water resource assessment and management.

## Description of the study site

**Fig. 1:**
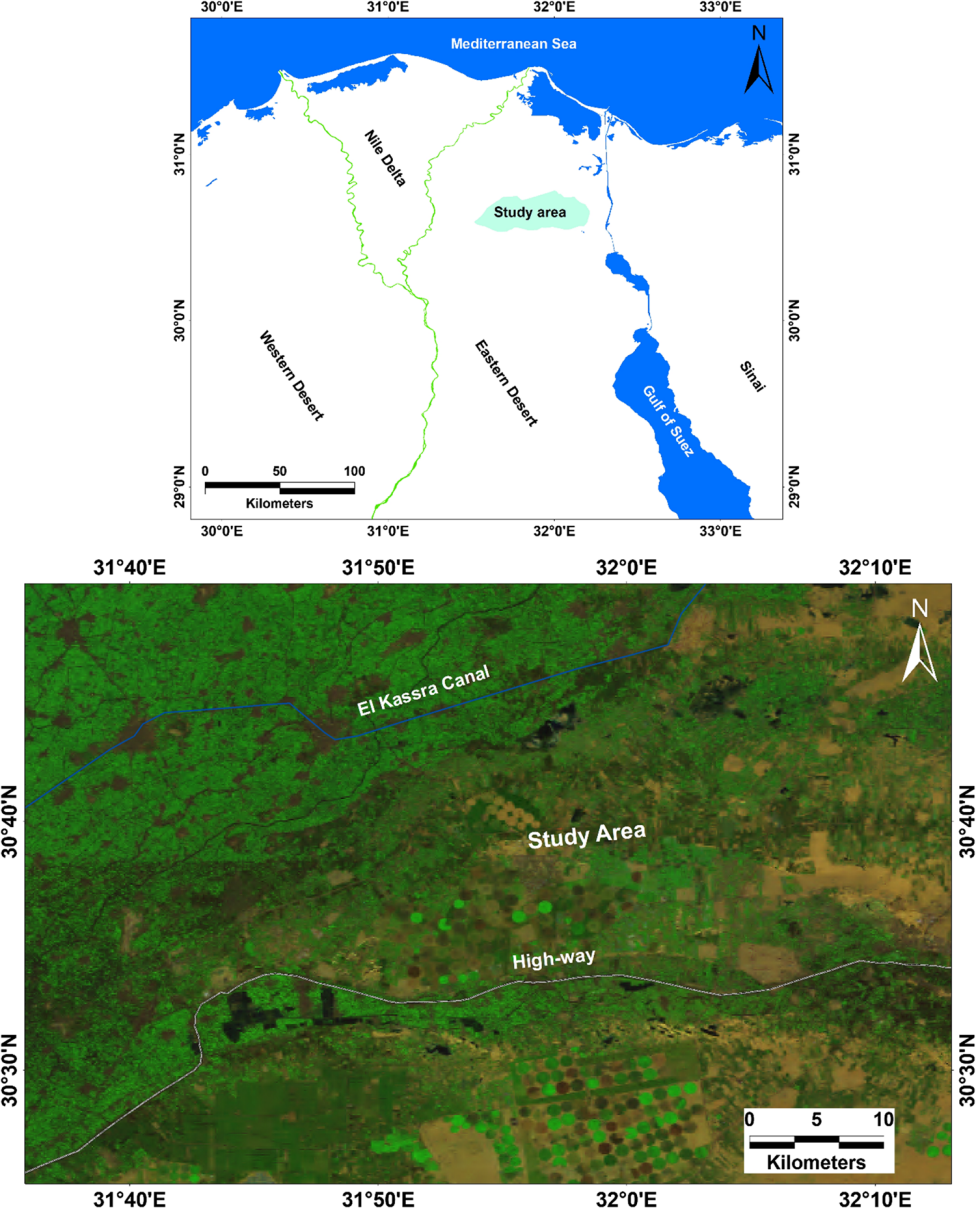
The study site location map

**Fig. 2:**
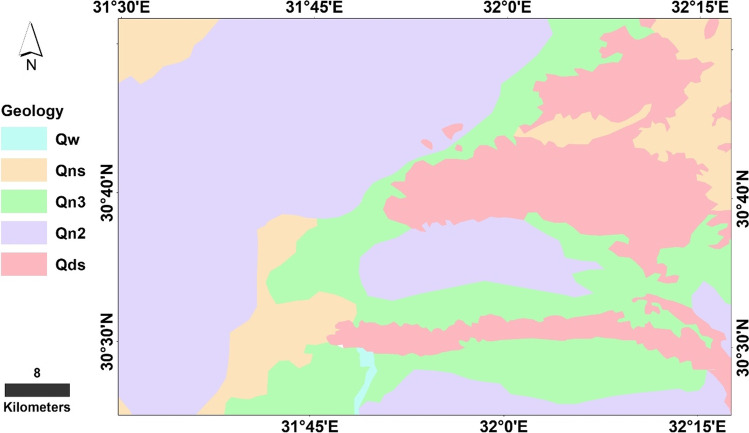
Lithological units in the study site

The study area is located to the east of the Nile Delta, and between El Kassara and Ismailia Canals, Egypt. It is bounded by latitudes 30° 29′ 24″ and 30° 44′ 12″ N and longitudes 31° 40′ 12″ and 32° 11′ 24″ E (Fig. 1). The area under consideration has been planned for great development including many of the reclaimed agricultural lands and industrial activities that are under construction. Moreover, the agricultural activities and irrigation systems have been irrigated using various water resource systems (surface water, groundwater, and conjunction of surface and groundwater). Geologically, the study area is occupied by rock units belonging to the tertiary and quaternary (Fig. 2). The Miocene sediments are dominated by clastic facies in the southern part of the study area; however, they changed into shallow marine sandy limestone and marls towards the north which consists mainly of alternating sandy-limestone, and loose-quartz sand and marl. The Eocene rocks underlying the Quaternary sediment are mainly composed of cracked and fissured carbonate rocks. Meanwhile, the northeastern portions (Pliocene sediments) are outcropped. The Pliocene clay is about 300 m thick and is overlain by the Quaternary deposits in the Nile Delta floodplain.

The Quaternary deposits which cover most of the study area are classified into two rock units: (1) the upper unit is the Holocene silt and clay of Nile (flood plain deposits) with Sabkha and fine sand deposits, while the lower one is (2) the Pleistocene gravels and sand. Some sand sheets and sand dunes were distributed in the area and of the Holocene age (Said and Beheri 1961; Shata 1965; El Fayoumy 1968; RIGW/WACO 1988). In the study area, the effect of the regional geological structures increases the thickness of the Quaternary aquifer by 3 m/km in the north direction (Gad 1995); it ranges from 300 to 400 m. Faults and folds are the most conspicuous structural elements system affecting the landscape in the study area. Normal faults are dominantly in the area represented by NE-SW and NW–SE directions. Along these faults, the vertical displacement varies from a few meters to hundred meters.

## Methodology

### WQI approach

**Fig. 3:**
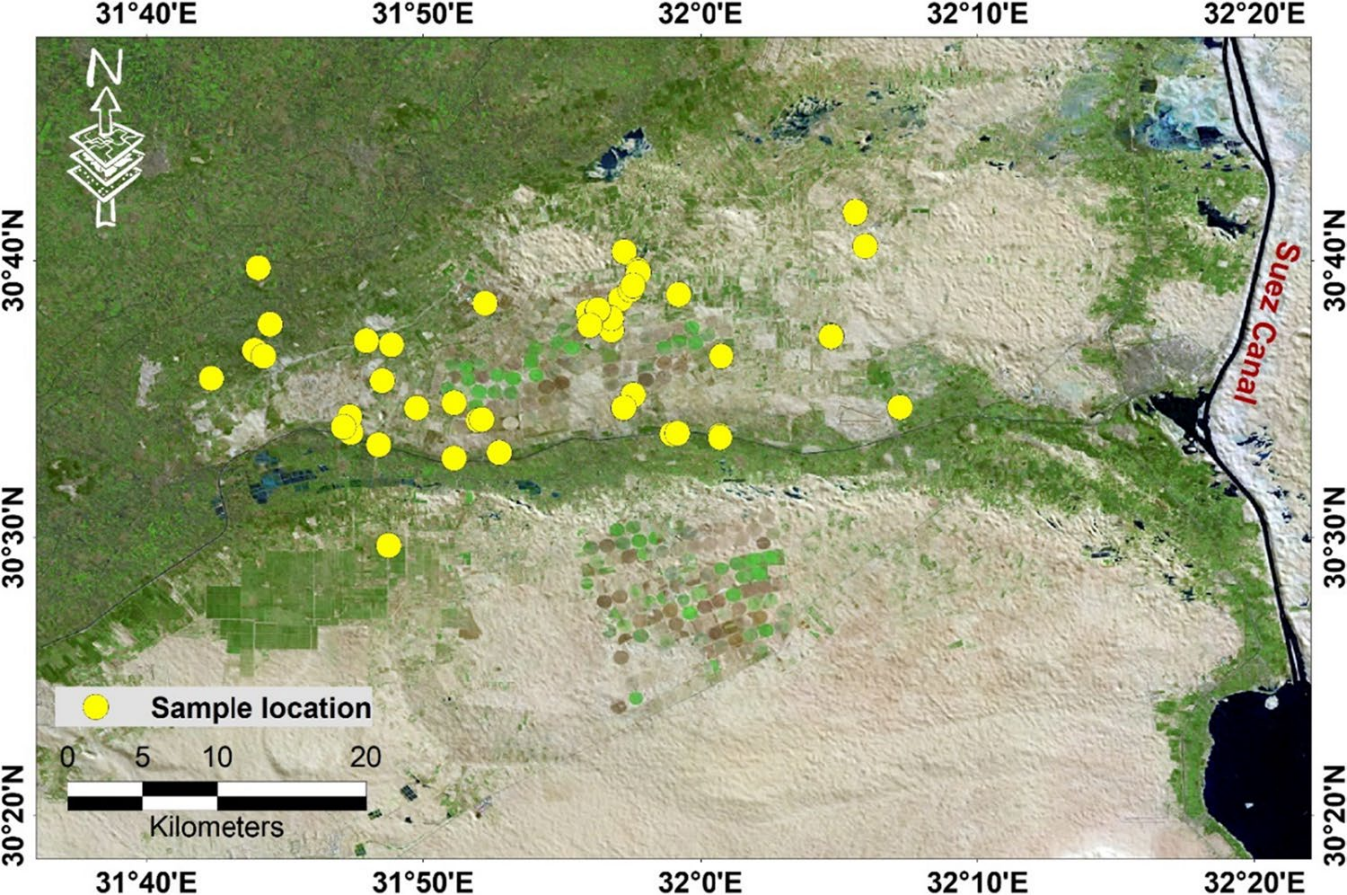
Site map of the groundwater samples collected during the present work

Sixty-seven groundwater samples were collected (Fig. 3) to construct water quality models and thematic layers. The water quality parameters are studied to investigate the suitability of groundwater for irrigation. The WQI was calculated based on previous studies (Sener et al. 2017; Soleimani et al. 2018; Suvarna, et al. 2020). In order to calculate WQI, the importance of water quality parameters poses into four steps including the following: (1) weight and numerical ranging were assigned to each group from 1 to 5, (2) the ratings were assigned for the selected individual parameters used in the study, (3) water quality parameter integration, and (4) classification of the resulted in water quality map into classes (excellent, good, moderate, permissible, and unsuitable). The weights of each index were assigned based on the following mathematical equation (Eqs. 1, 2) (Sener et al. 2017; Soleimani et al. 2018);

1 $$GWQI=\sum_{i=1}^{n}{W}_{i}\times {E}_{f}$$

2 $${W}_{i}={w}_{i}/\sum_{i=1}^{m}{w}_{i}$$

However, the term ${\mathrm{} w}_{i}$ refers to the weight of parameter *i*.

### WQI parameters

Basically, the water quality index (WQI) is the most effective tool to evaluate and quantify water quality in various regions (Kangabam et al. 2017; Karunanidhi et al. 2021a, b, 2022; Killivalavan et al. 2022; Panneerselvam et al. 2022). The excessive concentrations of dissolved ions in the water used for irrigation have a negative effect on the plants and agricultural soil both physically and chemically. The WQI applied in the present work is a useful index that describes the cumulative effect of the seven hazard groups on the quality of water used for irrigation. Accordingly, groundwater quality parameters such as salinity hazard, sodium percent sodium (%Na^+^), sodium hazard, permeability index (PI), residual sodium carbonate (RSC), magnesium ratio (MR), and Kelly ratio (KR) are more widely used for assessment of irrigation purposes (Table 1). However, the numerical effects of the specific values and units of various water quality parameters were transformed into a single value by an extant mathematical method (Cude 2001). The samples were collected from the site after 10–15 min of pumping; the pH, the electrical conductivity, (EC), and total dissolved solids (TDS) of each sample were measured in the field immediately after the sample was collected. The samples of groundwater were collected from wells and boreholes in the area of study. In the present work, 67 groundwater samples were collected and bottled in polyethylene bottles (500 ml), and they were stored in a 4 °C refrigerator. The chemical analysis was carried out in the Desert Research Center, Mattaria, Cairo, using various apparatuses and techniques. The analytical results of the collected samples are shown in (Table 2).

**Table 1:** Water quality indices for irrigation applied in this work

	Water quality indices	
Index	Formula	Eq. num
Sodium percentage	$Na\%=\left[\left({Na}^{+}+{K}^{+}\right)/\left({Ca}^{2+}+{Mg}^{2+}+{Na}^{+}+{K}^{+}\right)\right]\times 100$	(3)
Sodium absorption ratio	$SAR=\left[{Na}^{+}/\sqrt{\left({Ca}^{2+}+{Mg}^{2+}\right)/2}\right]\times 100$	(4)
Permeability index	$PI=\left[\left({Na}^{+}+{K}^{+}\surd Alkalinity\right)/\left({Ca}^{2+}+{Mg}^{2+}+{Na}^{+}+{K}^{+}\right)\right]\times 100$	(5)
Kelley index	$KI={Na}^{+}/\left({Ca}^{2+}+{Mg}^{2+}\right)$	(6)
Residual sodium carbonate	$RSC=\left(\left(Alkalinity\right)-\left({Ca}^{2+}+{Mg}^{2+}\right)\right)$	(7)
Magnesium hazard	$MH={Mg}^{2+}/\left({Ca}^{2+}+{Mg}^{2+}\right)$	(8)

**Table 2:** Statics of the chemical analysis of the collected samples

Parameter	Range	Minimum	Maximum	Mean	Std. error mean	Std. deviation
EC	4682.00	300.00	4992.00	2876.48	172.63	1244.86
pH	0.97	7.56	8.54	7.84	0.03	0.20
TDS	3752.00	173.00	3913.00	1733.69	113.76	827.62
Ca^2+^	259.80	3.50	263.10	97.82	9.46	68.21
Mg^2+^	79.87	1.09	80.96	35.39	2.74	19.77
Na^+^	898.67	23.47	922.14	487.91	30.56	220.34
K^+^	16.80	2.08	18.85	8.37	0.46	3.31
CO_3_^2−^	76.50	0.00	76.50	21.07	1.63	11.78
HCO_3_^−^	335.93	124.44	460.33	260.64	9.77	70.48
SO_4_^2−^	1173.42	25.24	1198.76	419.54	39.25	283.06
Cl^−^	1282.56	15.71	1308.36	533.24	40.25	290.21

### SVM and SVM-WQI models

Support vector machine (SVM) is a supervised algorithm of machine learning which can be used for classification or regression. However, mostly, SVM is used for classification challenges. The SVM approach deals with a binary classification model that assumes there are two classes $C={c}_{1},{c}_{2}$ of an object belonging only to one of these classes. Basically, SVMs were developed for binary classification that uses hyper-planes to identify the decision boundaries between the data points of various classes (Luisa et al. 2010). Thus, a suitable kernel function is used in the SVM approach to translate the original data points from the input space into a high-dimensional or even infinite-dimensional feature space, where a maximal separation plane (SP) is created. The kernel function selection is generally dependent on the data distribution. However, the kernel function according to Widodo et al. (2007) is usually selected through a “trial and error” method. Linear, sigmoid, polynomial, and radial basis functions are the possible choices of kernel functions. Radial basis function (RBF) is the most common kernel functions applied in most research applications and concluded in Eq. (9); it has the form (Xie et al. 2008).

9 $$K({x}_{i},{x}_{j})=\mathrm{exp}(-\gamma {\Vert {x}_{i}-{x}_{j}\Vert }^{2})$$

where $\gamma$ (gamma) controls the decision boundary smoothness in the feature space. The SVM classification model with RBF was applied to the groundwater quality data in this paper.

**Fig. 4:**
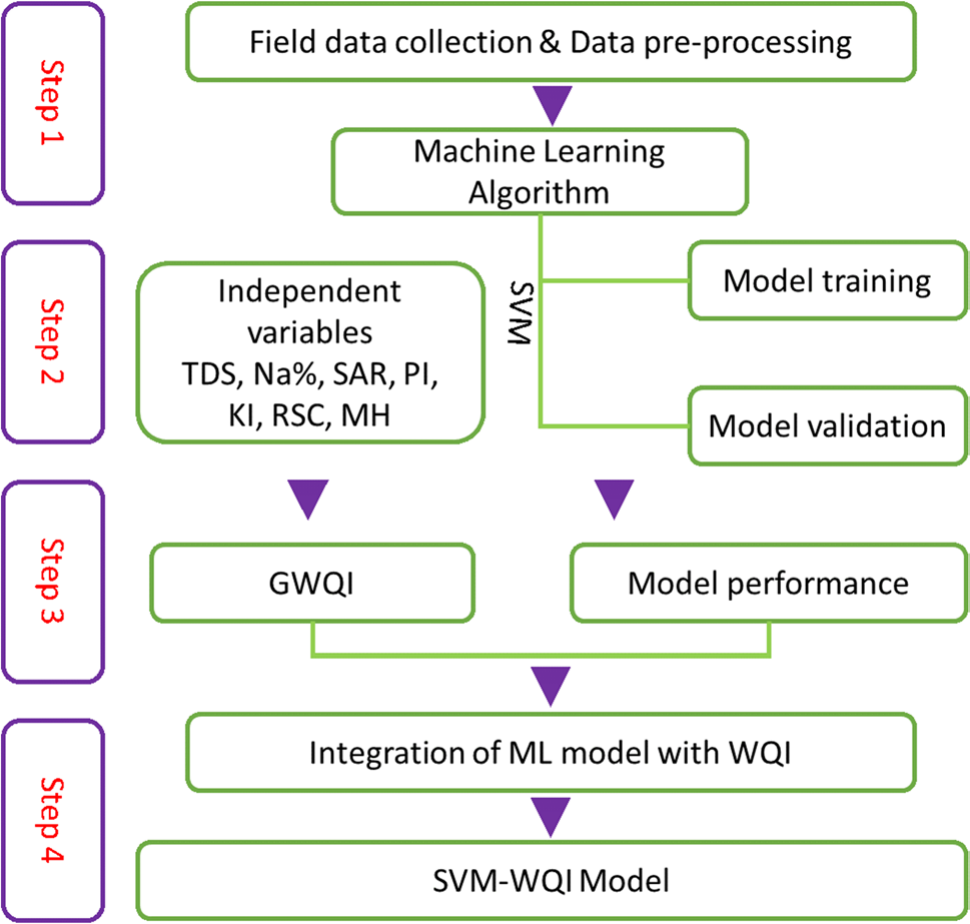
Flowchart of the methodology

Support vector machine (SVM) automatically will map the training data into a featured space as a machine learning approach. However, the WQI approach as a statistical method depends on weights, rating, and classification. Therefore, the SVM-WQI model, integrates the two approaches result for building a new groundwater assessment model. Therefore, in order to achieve the integration, stacking was employed in the programming environment. Stacking facilitates mapping over multiple sub-classes (dimensions), and the layering, then the map layer stacking will be revealed. Figure 4 represents the flowchart of methodology in the present work.

## Results and discussions

### Independent variables

#### Salinity index and pH

In most cases, salinity exists when salt accumulates in the crop root zone that is associated with or strongly causes a loss in yield. Salts that contribute to salinity are frequently water-soluble and are transported by water. Accordingly, the salinity problems reduced the infiltration which led to letting the water remain too long on the soil surface. In the present study, the TDS was found within the range of 173 to 3913 mg/l. Therefore, high TDS values over 2000 mg/l, which is higher than the recommended by FAO (1978a, b), led to severe problems. The results of salinity values revealed that about 42% of the collected samples belonged to high salinity values. The average pH values were measured between 7.56 and 8.54, and most of the samples were within the recommended limit according to FAO (1978a, b). Generally, some problems can occur in water with pH values 8 and above with a high concentration of bicarbonates. The measured electrical conductivity values ranged from 300 to 4994 uS/cm that exceeded the standard recommended by FAO (1978a, b). According to Handa (1969) classification of EC (Table 3), the water samples belonged to high to very high classes. Therefore, plants and agricultural activities capable of high salinity should be applied.

**Table 3:** Water classification based on the EC values (μS/cm) (Handa 1969)

EC (μS/cm)	Water salinity	Range (study site)	Percentage
0–250	Low salinity (excellent quality)	–-	–-
251–750	Medium salinity (good quality)	300–627	5.77%
751–2250	High salinity (permissible quality)	768–2238	26.92%
2251–6000	Very high salinity	2262–4992	67.31%
6001–10,000	Extensively high salinity	–-	–-

#### Sodium percentage (% Na^+^)

The sodium percent (% Na^+^) can be expressed in Eq. 3 in percentage. The increase of Na^+^ percent could reduce the soil permeability (Todd 1980; Doneen 1964; Sundaray et al. 2009); thus, the soil required special treatment to increase its permeability to help plants grow. However, the sodium ion concentration in the groundwater samples ranged from 23.46 to 922.13 mg/l. High concentration levels of sodium ions were observed mainly southeast of the study site. This increase is normally attributed to the increase in chloride ions, which contributed to the brackish water intrusion with groundwater. The Na^+^ percent values across the collected samples ranged from 40.05 to 96.35 (Table 4). Therefore, the majority of the collected groundwater samples (about 86%) were located between doubtable and unsuitable water classes for irrigation.

**Table 4:** Quality index statistics of the collected samples

		Quality index statistics				
Stats	RSC	SAR	Na %	PI	KI	MH
Min	105.26	1.07	40.05	60.87	0.56	28.93
Max	436.89	21.56	96.35	123.95	26.16	64.68
Median	231.32	11.43	73.53	81.09	2.75	37.25

#### Sodium absorption ratio (SAR)

**Fig. 5:**
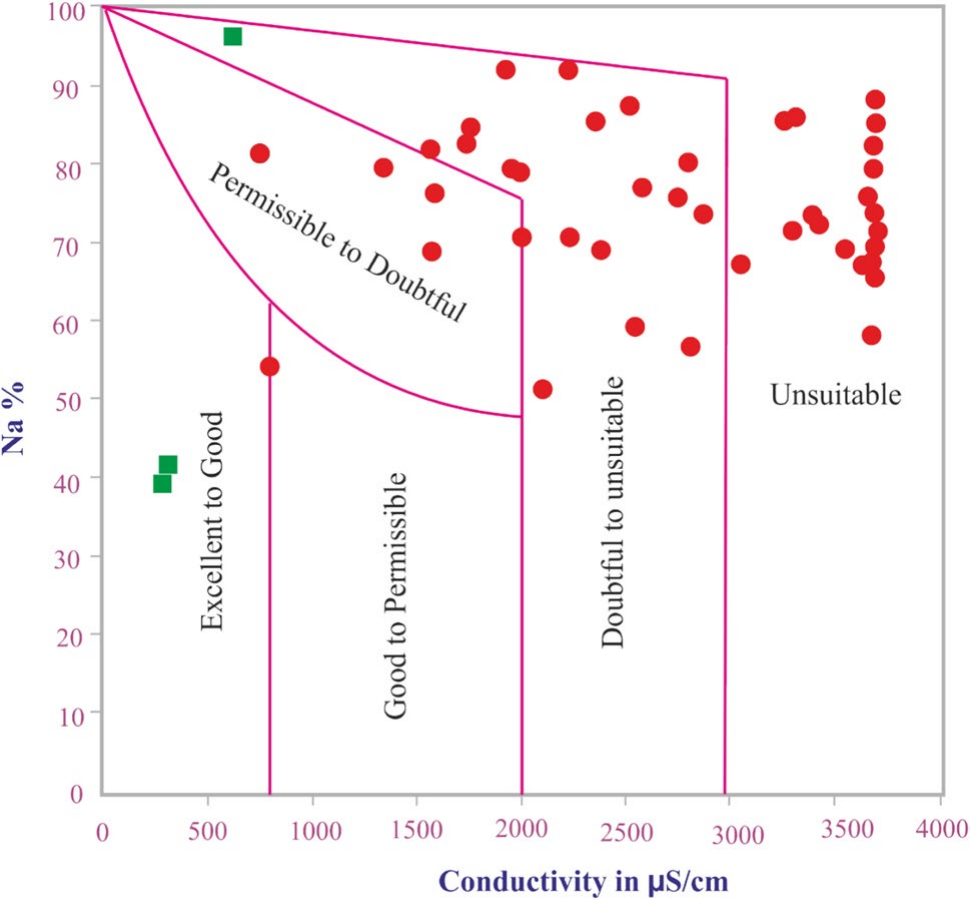
Vilcox diagram of the collected samples

The SAR is more contributed to the soil sodium exchangeable percentage and can be expressed in Eq. 4 in Table 1. Moreover, the aggregates’ stability and hydraulic conductivity were found affected by the increase in the SAR percentage (Suarez et al. 2006). According to Eq. (4), the SAR values of the study site samples ranged from 1.07 to 21.56 (Table 4) indicating that most of the samples were located between excellent to good water classes. The Na % value was plotted against the EC to assess the irrigation waters (Fig. 5). Plotting the collected groundwater samples on the Wilcox diagram (Wilcox 1955) revealed that approximately, the majority of the samples are in the class of doubtful-unsuitable to unsuitable. About 9% (5 samples) were in the category permissible-doubtful class. The results from the Vilcox diagram stated that the collected groundwater samples are doubtful-unsuitable for irrigation purposes.

#### The permeability index (PI)

Since the permeability of the soil was influenced by the concentration levels of Na^+^, Mg^2+^, Ca^2+^, and HCO_3_^−^, the PI was developed in 1964 (Doneen 1964) and can be calculated as Eq. 5. Waters based on the values of the PI can be categorized into three categories: I, II, and II (> 75%, 75–25, and < 25%) respectively. The permeability index across the collected samples ranged from 60 to 123. According to the Doneen chart in the year 1990 (Domenico and Schwartz 1990), all collected samples were located in class I and class II. Thus, for the permeability index values, 75% of the groundwater samples fall under class I (PI > 75%), and the remaining 25% belong to class IIranging from 25 to 75%).

#### Kelly index (KI)

KI was developed by Kelly (1951) and can be expressed mathematically using the equation proposed by Kelly (Eq. 6). Kelly index was applied to evaluate the suitability of water for irrigation purposes; in this way, the KI deals with the evaluation of the excess sodium in water (Kelly 1940, 1951). Generally, values more than unity (1) indicate an excess of sodium with unsuitable water for irrigation. Therefore, values of KI less than 1 demonstrate water suitability for irrigation purposes. Herein, the KI of the collected samples varies from 0.56 to 26.16 with an average of 3.82 (Table 4), representing the unsuitability of groundwater.

#### Sodium residual carbonate (RSC)

With increasing bicarbonate concentration, Ca^2+^ and Mg^2+^ were precipitated as carbonates. This impact of bicarbonate and Ca^2+^ and Mg^2+^ in water was expressed using Eq. 7 in Table 1. The sodium residual carbonate (RSC) formula was developed by Ragunath (1987), allowing the assessment of the quality of water for irrigation. The values of the RSC in the collected groundwater samples ranged from 105 to 438 with an average value of 240 (Table 4). According to the results, values of RSC indicate that all the collected samples in the study area are of unsuitable water class for irrigation. Therefore, the suitability of groundwater relies on the results of the alkalinity excess more than the sum of the Mg^2+^ and Ca^2+^ concentrations in water.

#### Magnesium hazard (MH)

According to Wilcox (1955) and Hem (1985), the Mg^2+^ and Ca^2+^ ion concentration maintains the equilibrium condition in groundwater. The increase in Mg^2+^ concentration could affect adversely the soil quality, resulting in a decrease in crop yield. Magnesium hazard can be expressed in Eq. 8; MH less than 50 can be used safely for irrigation. The values of MH (Table 4) ranged from 28.93 to 64.68. Most of the samples (85%) in the study site were of low MH.

### Hydrochemical facies

#### Correlation analysis

**Fig. 6:**
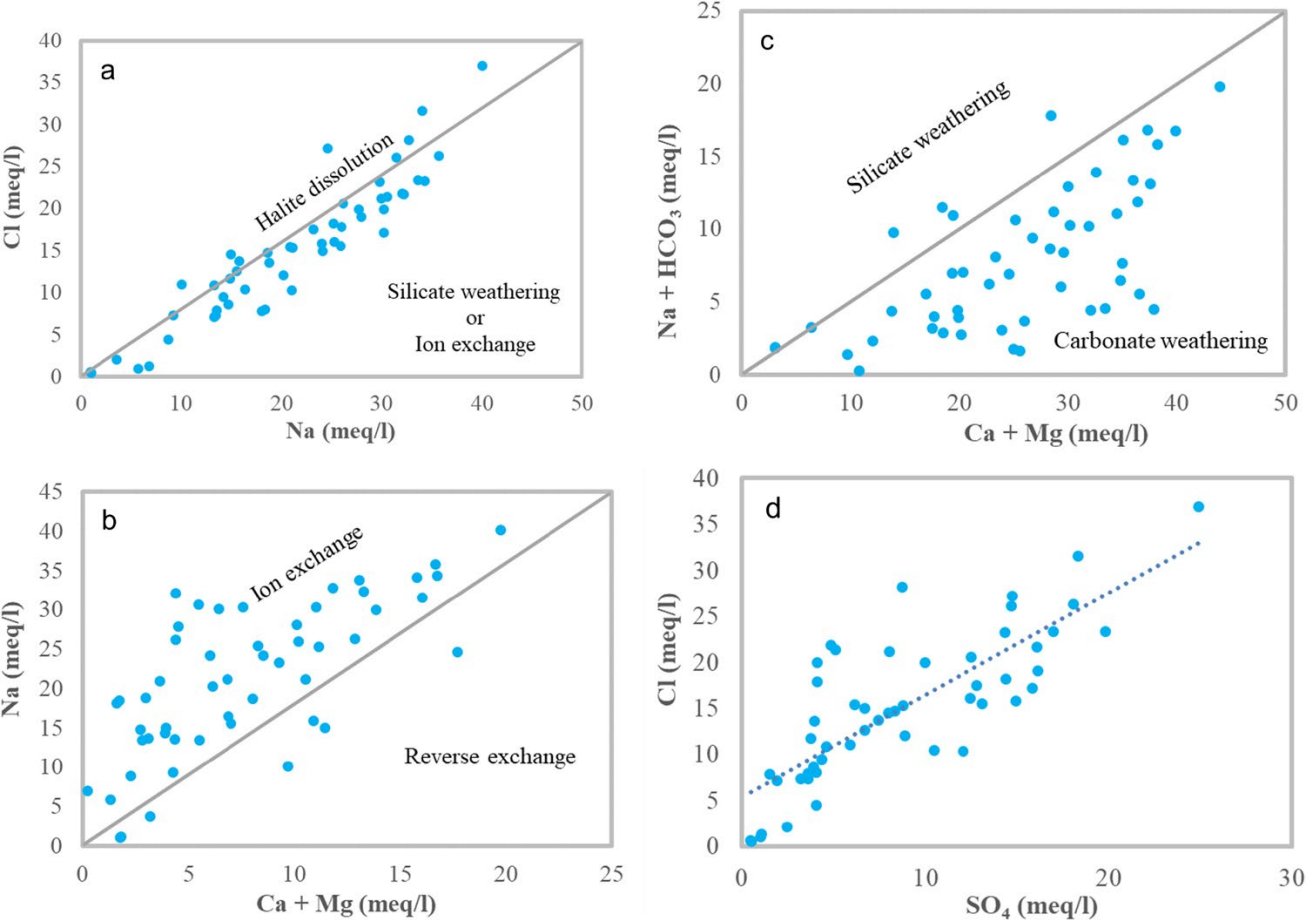
Hydrochemical relationships of the collected samples

The relation of several cations and anions that can be determined by the water facies can be used. Among these relations are (Na^+^ vs. Cl^−^), (Na^+^ vs. Mg^2+^  + Ca^2+^), (Na^+^  + HCO_3_^−^ vs. Mg^2+^  + Ca^2+^), and (Cl^−^ vs. SO_4_^2−^) (Table 5) (Masoud and Abu El-Magd 2022). In general, the relationship between sodium and chloride is important for determining the pathways of water salinity. Initially, most of the collected groundwater samples fall below the halite dissolution line (Fig. 6a) having a high correlation coefficient (*R*^2^ = 0.86). Furthermore, the relation of (Na^+^ vs. Ca^2+^  + Mg^2+^) in figure (Fig. 6b) many of the samples (89%) fall below the equimolar line with a strong determination coefficient (*R*^2^ = 0.52). These relationships suggest ion exchange or silicate mineral weathering (Fig. 6a and b). The plot of collected samples on (Na^+^  + HCO_3_^−^ vs. Ca^2+^  + Mg^2+^) the graph indicates that most of the samples fall below the equimolar line, suggesting carbonate weathering (Fig. 6c). Figure 6d represents the relation between (Cl^−^ vs. SO_4_^2−^); the plot indicates a positive relationship with *R*^2^ = 0.62.

**Table 5:** Statistics of hydrochemical ratios in the study site

Stats	meq/l					Cl^−^/anions
	Mg^2+^/Ca^2+^	Na^+^/Cl^−^	Na^+^/K^+^	Cl^−^/SO_4_^−^	(Ca^2+^ + Mg^2+^) / (K^+^ + Na^+^)	
Min	0.41	0.91	3.58	0.81	0.04	0.14
Max	1.83	6.14	346.47	5.04	1.50	0.67
Geomean	0.67	1.55	90.23	1.78	0.33	0.46
Average	0.72	1.67	112.97	2.00	0.41	0.49

#### Index of base exchange

A comprehensive parameter to measure and describe the reactions in groundwater is the index of base exchange (IBE) that was developed (Schoeller 1965). The IBE is chloro-alkaline indices, i.e., CA1 and CA2, that are applied to measure the extent of base exchange during water–rock interaction. Huang et al. (2018) interacted with the importance of chloro/alkaline indices to study the water cation exchange process. The IBE can be mathematically calculated by the following expression CA1 and CA2, where all ion concentrations applied in this formula (Eq. 10, 11) are expressed in (epm):

10 $$CA1=\left[Cl-\left(Na+K\right)/Cl\right]$$

11 $$CA2=\left[Cl-\left(Na+K\right)/\left({SO}_{4}+{CO}_{3}+{HCO}_{3}+{NO}_{3}\right)\right]$$

When the above index results are negative, there is an expectation of an exchange of Mg^2+^ or Ca^2+^ in groundwater with K^+^ and Na^+^ in the aquifer material. However, positive values of the above indices indicate a reverse ion exchange (Schoeller 1965, 1967). Calculating the above indices for the groundwater samples reveals that more than 96% of the collected groundwater samples in the site exhibit ion exchange of Ca^2+^  + Mg^2+^ to K^+^  + Na^+^ in groundwater which results in the enhancement of K^+^  + Na^+^ ions in groundwater.

### Groundwater quality index (GWQI)

**Fig. 7:**
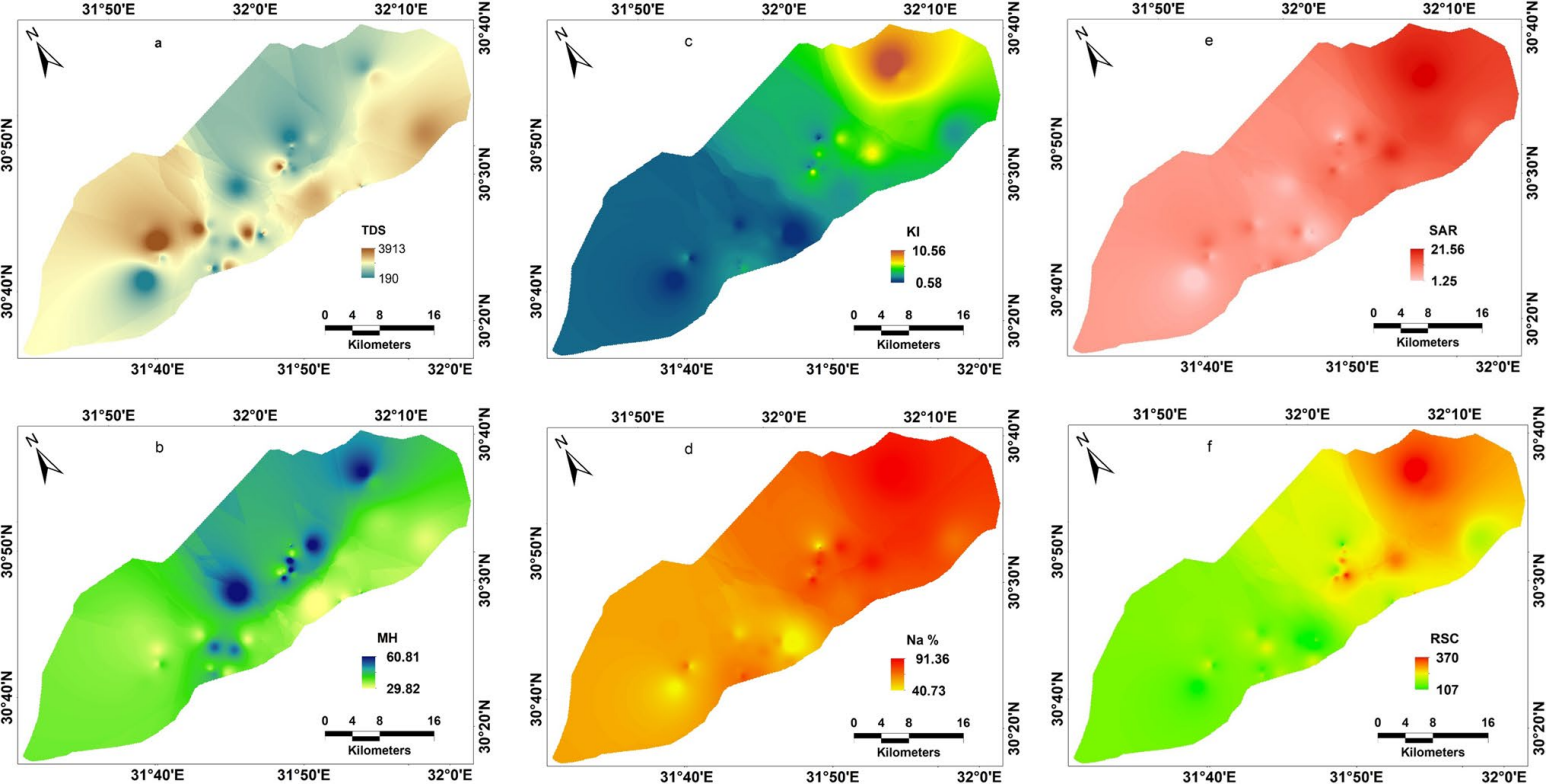
Groundwater indices; **a** TDS; **b** magnesium hazard; **c** KI, **d** Na%; **e** SAR; and **f** RSC

**Fig. 8:**
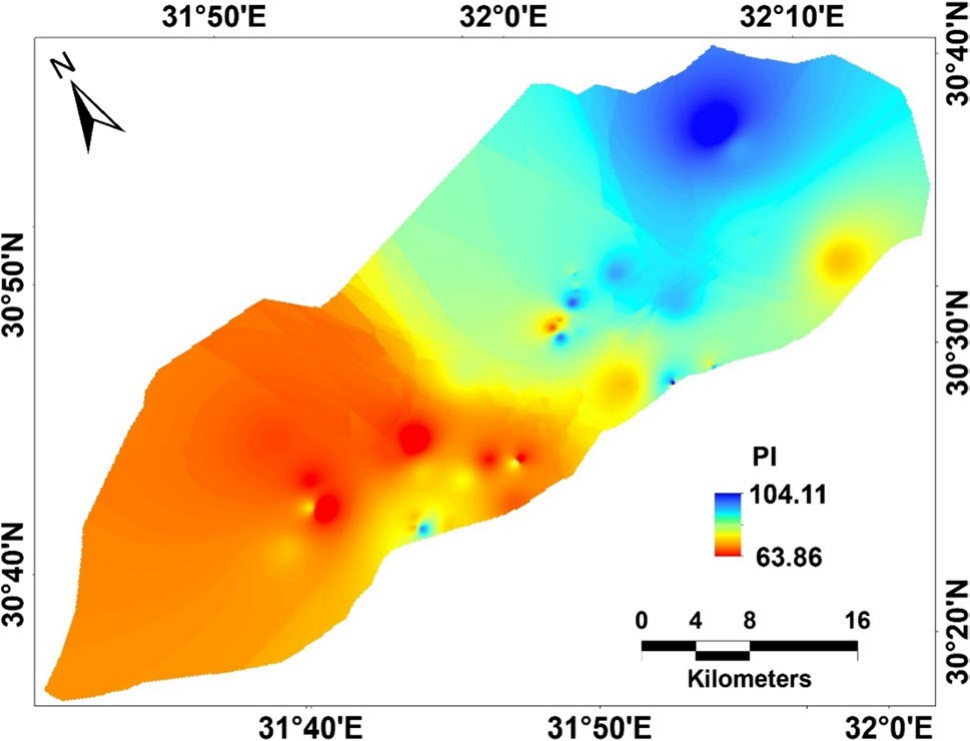
Permeability index map

The GWQI approach was implemented in this work, for the evaluation of groundwater quality for irrigation uses in the El-Salhyia area. The applied approach in the current work is considered the simplest method to provide grading for water quality by using a list of hazard factors and their concentration. Seven parameters were calculated from the hydrochemical results of the collected groundwater samples. The suitability of groundwater for irrigation was estimated according to the cumulative effects of these seven parameters. Mathematically, the map weight and the groundwater quality index can be expressed as the following equations (Eqs. 12 and 13).

12 $$GWQI=\sum_{i=1}^{n}{W}_{i}\times {E}_{f},$$

13 $${W}_{i}=\sum TDS+{M}_{h}+{K}_{i}+{P}_{i}+{S}_{AR}+{N}_{a\%}+{\mathrm{R}}_{SC},$$

where *W*_*i*_ denotes map weight, $TDS$ denotes the total dissolved solids, ${M}_{h}$ denotes the magnesium hazards, ${K}_{i}$ denotes the Kelly index, ${P}_{i}$ denotes the permeability index, ${S}_{AR}$ denotes sodium absorption ratio, ${N}_{a\%}$ donates the sodium percentage, and ${R}_{SC}$ denotes residual sodium carbonate.

where $GWQI$, groundwater quality index; W_i_, map weight, and ${E}_{f}$, efficiency value. However, the map classes and the efficiency values can be shown in Table 6. The thematic layers (Figs. 7 and 8) with their efficiency were integrated in the GIS platform to produce the final GWQI map, for agriculture purpose management. The calculation of the hazard factors used to estimate GWQI can be calculated by mathematical expressions listed in Table 1.

**Table 6:** Thematic map classes and efficiency value

Category	classes and efficiency		Efficiency value
	Range	Class	
EC	< 250	Excellent class	0.40
	250–750	Good class	0.30
	750–2250	Permissible class	0.20
	> 2250	High class	0.10
SAR	< 10	Excellent class	0.44
	10–18	Good class	0.33
	18–26	Doubtful class	0.22
PI	> 75	Good-class 1	0.50
	25–75	Good-class 2	0.33
	< 25	Unsuitable class	0.17
RSC	< 1.25	Good class	0.50
	1.25–2.5	Doubtful class	0.33
	> 2.5	Unsuitable class	0.17
Na %	< 40	Excellent class	0.40
	40–60	Good class	0.30
	60–80	Doubtful class	0.20
	> 80	Unsuitable class	0.10
KI	< 1	Good class	0.75
	> 1	Unsuitable class	0.25
MH	< 50	Good class	0.75
	> 50	Unsuitable class	0.25

**Fig. 9:**
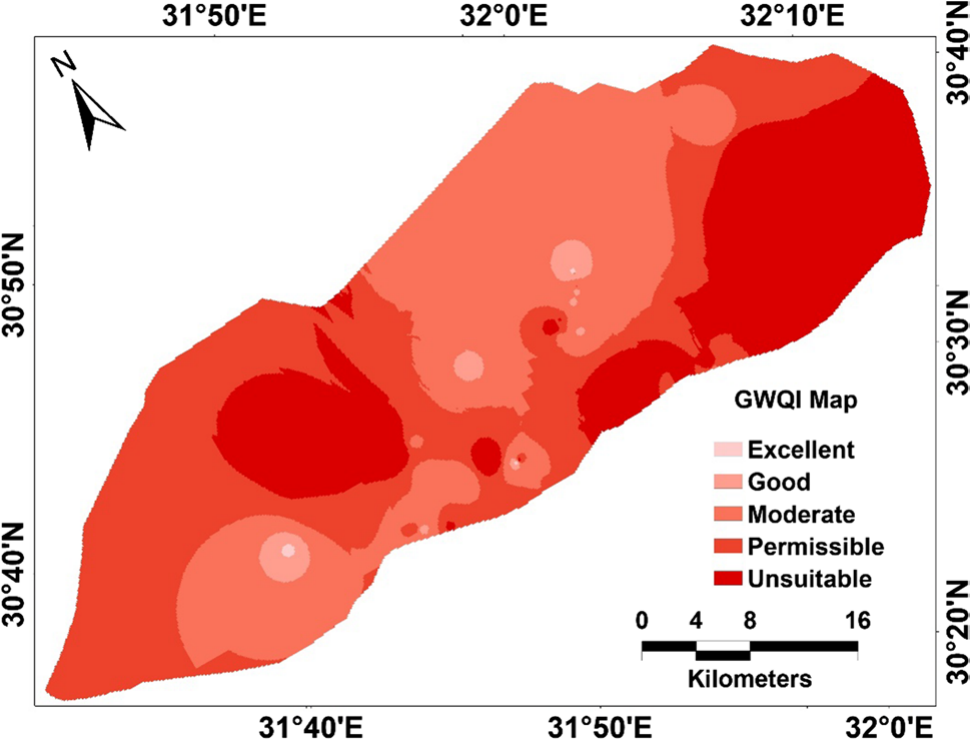
Groundwater quality index mapping (GWQI) of the study site

For the weights and efficiency, higher weight was given to the higher importance of the indices in calculating GWQI. Accordingly, the higher efficiency value was assigned to the excellent to good water quality class. The GWQI distribution map over the area reflects a wide range of variations in quality. Furthermore, the final GWQI map (Fig. 9) evaluates the overall water quality in the study site, indicating that the eastern and northern-middle areas are of unsuitable groundwater quality. The unsuitability of the eastern part for irrigation possibly contributed to the ion exchange and/or saltwater intrusion. The implementation of the GWQI and multivariate statistical approaches is an effective tool providing a better understanding of groundwater quality for assessment for irrigation.

### SVM model

The SVM model was trained and tested in the (Jupyther) tool using the anaconda environment (www.anaconda.com). The SVM technique was applied for the spatial classification of the assessment of groundwater quality. The complete groundwater quality dataset was divided into two subsets (training, and validation or test). Before running the ML model (SVM), collected data was divided into two datasets classes the training dataset (70%) and the testing or validation dataset (30%). The spatial SVM was performed on a dataset comprised of seven features (TDS, Na%, SAR, PI, KI, RSC, MH). Among the SVM kernel functions, the RBF kernel functions were selected in the SVM model. In the spatial classification model, the SVM technique was employed to differentiate between the five classes or categories of sampling in the site. These classes include excellent quality, good quality, moderate quality, permissible, and unsuitable groundwater quality.

**Fig. 10:**
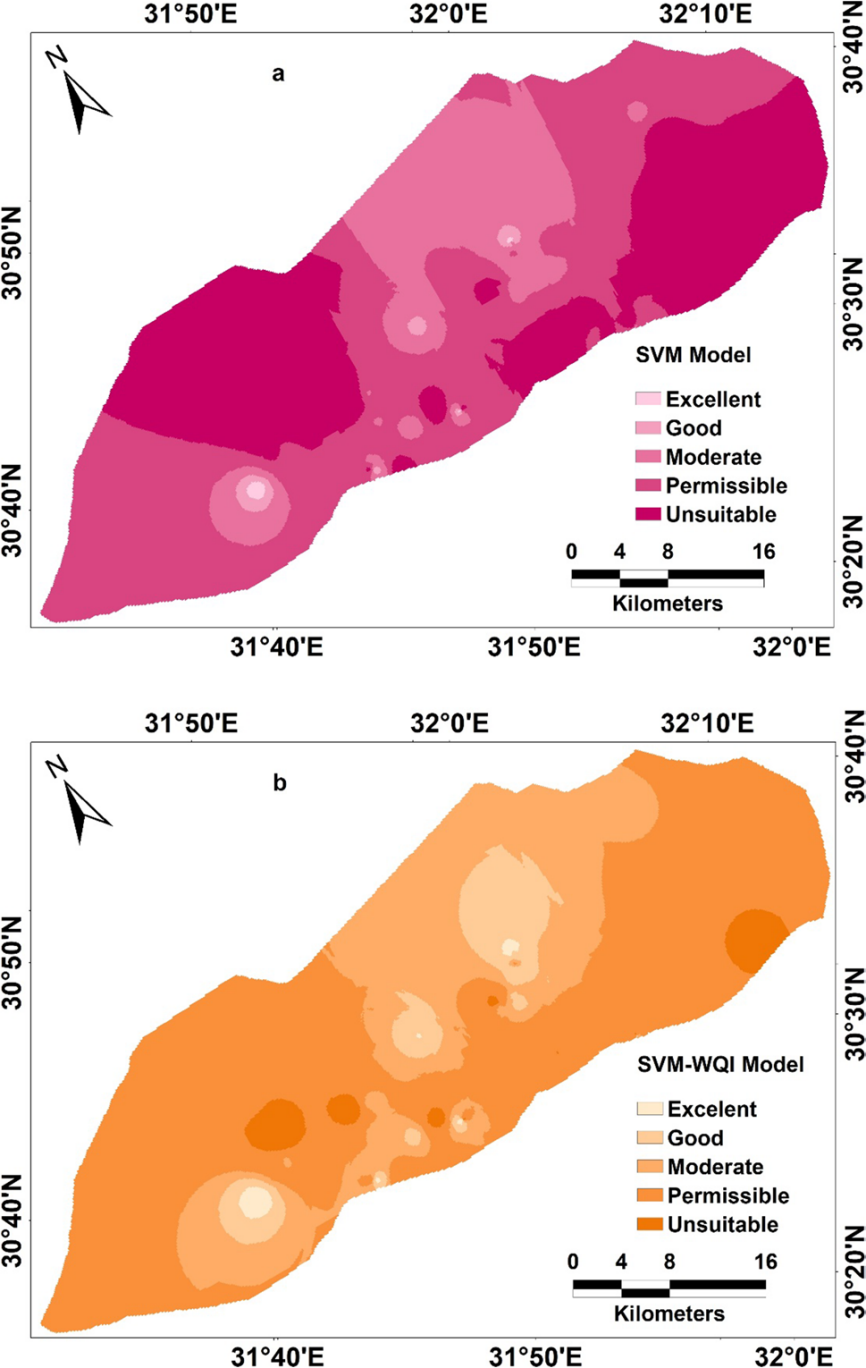
Machine learning models, a SVM model and b SVM-WQI model

SVM with RBF kernel function from the results shows a better prediction of groundwater quality mapping as compared to the GWQI mapping. Where, permissible and unsuitable class values range from 36 to 27% and 45 to 36% for the WQI approach and SVM method respectively (Table 7). The SVM model was successfully trained automatically after the selection of the type of support vector machines kernel function, and then the model was trained with seven predictors. This could be since the RBF kernel function is able to capture the behavior of the model accurately more than the GWQI approach. The result of the SVM model with seven input predictors is shown in Fig. 10a and Table 7 respectively. As shown in Table 7, the percentage of the unsuitable area from the SVM model was decreased compared to the same class of the GWQI model.

**Table 7:** Percentage of the area comprises by each class

Model	Excellent	Good	Moderate	Permissible	Unsuitable
GWQI	0.2%	2%	34.8%	36%	27%
SVM	1%	1%	18%	44%	36%
SVM-WQI	1%	2%	14%	68%	15%

The integrated approach of the support vector machine and water quality index approach (SVM-WQI) was applied here, to assess groundwater. They found that the SVM-WQI method provided better estimation results than individuals of SVM and WQI. The results are shown in Fig. 10b, and the analysis results of the model assessment for different classes of groundwater quality are shown in Table 7. Similarly, the SVM-WQI model shows a low percentage of the area for excellent class compared to the SVM model and WQI mapping. However, the accuracy of the SVM-WQI model was determined to be 0.90, since the majority of calculated hazard indices shows that most of the samples lie in permissible and unsuitable groundwater quality area reached which matched with the results from the SVM and SVM-WQI models. Furthermore, the present study clearly noted that when the model was applied towards WQI, SVM, and SVM-WQI, the accuracy of the model prediction will significantly increase as shown for both using SVM and SVM-WQI models.

#### Variable importance

**Fig. 11:**
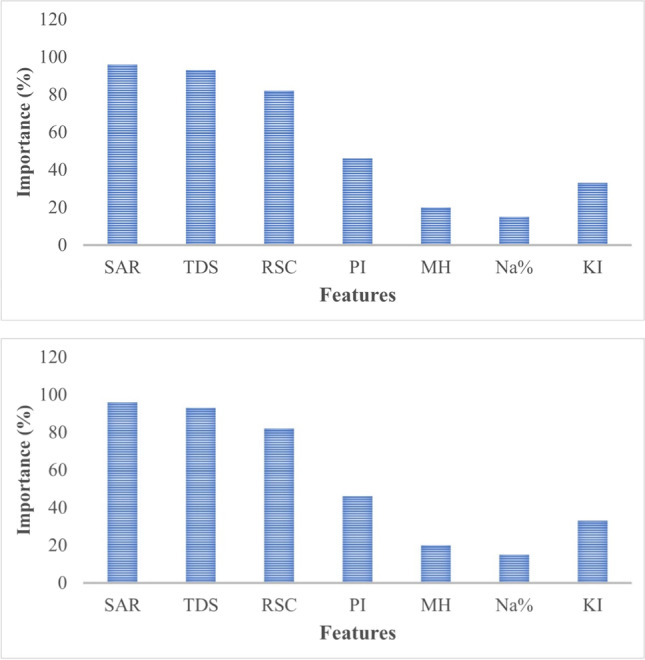
Variables importance for the SVM model

The chosen independent variables for the SVM model are implemented using variable importance (Fig. 11). According to Jardine et al. (2006), the removal of the irrelevant variables from the dataset is required prior to the classification modeling. The presence of such irregular features would lead to a misfit of the model, thus losing the ability of prediction. The calculated parameters of the hydrochemical dataset (Na%, SAR, PI, KI, RSC, MH) with TDS were used as independent variables for the model. Figure 11 illustrates the importance of features in the spatial classification using the SVM model. It may be noted that SAR followed by TDS and then followed by RSC were the most important variables in classification, whereas the Na% followed by magnesium hazards were the most less-important variables in the spatial classification of the groundwater quality. This may be attributed to the fact that the SAR, TDS, and RSC largely determine the suitability of groundwater for irrigation. Furthermore, the TDS as an anthropogenic parameter in the study area largely determines the groundwater quality.

#### Model validation and accuracy

Hyper-parameter setting (*γ*) and (*ε*) as kernel parameters are mostly considered to be quite vital in shaping SVM model generalization performances. The ε-RBF is implemented for predictions, so the *γ* is set to 7, with *ε* values ranging from 0.001 to 0.07. It is noted that the MSE increases with an increase in values of (*ε*) while decreases in the numbers of support vectors were observed. Furthermore, *ε* value of 0.001 yields acceptable numbers of support vectors with minimal generalization errors. Table 8 represents the model accuracy for the applied approaches (SVM and SVM-WQI).

**Table 8:** Model accuracy for SVM and SVM-QWI

Dataset	Sensitivity (%)	Specificity (%)	Accuracy (%)
SVM	77.13	84.89	88.79
SVM-WQI	76.32	85.88	90.6

**Fig. 12:**
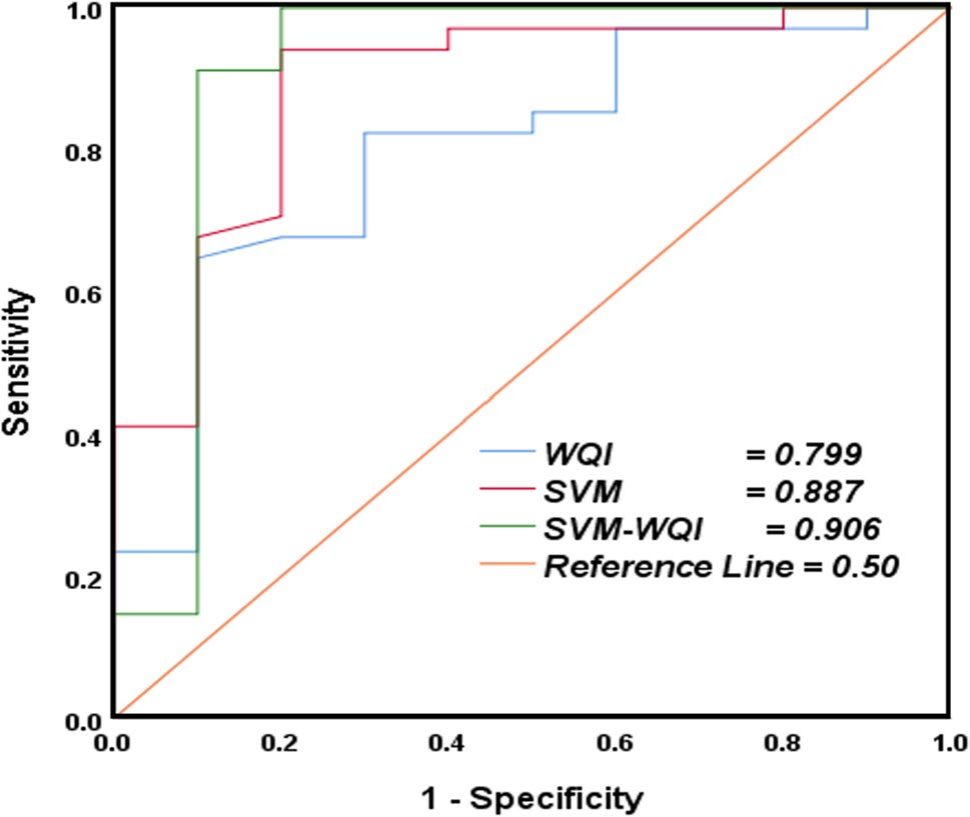
Receiver operating characteristics (ROC) curve for WQI, SVM, and SVM-WQI models

Furthermore, the classification performance according to the AUC (receiver operating characteristics (ROC)) curve was greater than 0.5, which indicates that, for all points on the curve, the true positive rate (sensitivity) was greater than false positive rates (1–specificity), thus showing satisfactory results. Therefore, it is evident that the results of the area under the curve (AUC) curve of SVM-WQI are more precise in accuracy as compared to WQI and SVM stand-alone approach (Fig. 12).

## Conclusions

The evaluation and prediction of groundwater quality are very substantial in managing pollution for sustaining the availability of freshwater resources. Herein, the SVM approach with RBF function integrated with WQI (SVM-WQI) was introduced to estimate the groundwater quality. The developed model in this context accurately estimated the groundwater quality using seven independent variables with relatively minor errors and proved a quite robust performance.

- The ion ratio relationships, diagrams, and correlation matrix indicating the dominance of the hydrochemical process in the area may be related to the dissolution process through water–rock interaction and the effect of leaching and dissolution.
- Referring to correlation, a positive and good correlation degree was observed between Ca^2+^, Mg^2+^, and Na^+^ with Cl^−^ and SO42^–^. The higher correlation degree supports the exchange process and the effect of leaching and dissolution intrusion.
- The SVM-WQI model shows a low percentage of the area for excellent class compared to the SVM model and WQI mapping.
- The presented SVM and SVM-WQI model performance was investigated using mean square error (MSE) with values ranging from 0.002 to 0.41 and accuracy values ranging from 0.88 to 0.90; hence, the SVM-WQI model revealed the highest accuracy.
- The integrated approach was classified into five categories: excellent, good, moderate, permissible, and unsuitable classes.

However, the finding in the present work seems to be reasonable; the application of groundwater quality variables is so far sensitive. It is suggested to explore further investigation of optimal other quality indices.

## Data Availability

The data that support the findings of this study are available from the corresponding author, upon reasonable request.
